# Morphological and Functional Changes in Skin of Adult Male Rats Chronically Treated with Letrozole, a Nonsteroidal Inhibitor of Cytochrome P450 Aromatase

**DOI:** 10.1267/ahc.20009

**Published:** 2020-09-08

**Authors:** Kamila Misiakiewicz-Has, Alicja Zawiślak, Anna Pilutin, Agnieszka Kolasa-Wołosiuk, Paweł Szumilas, Ewa Duchnik, Barbara Wiszniewska

**Affiliations:** 1 Department of Histology and Embryology, Pomeranian Medical University in Szczecin, 72 Powstańców Wielkopolskich Str., 70–111 Szczecin, Poland; 2 Department of Interdisciplinary Dentistry, Pomeranian Medical University in Szczecin, 72 Powstańców Wielkopolskich Str., 70–111 Szczecin, Poland; 3 Department of Social Medicine and Public Health, Pomeranian Medical University in Szczecin, 48 Żołnierska Str., 71–210 Szczecin, Poland; 4 Department of Dermatology and Venereology, Pomeranian Medical University in Szczecin, 2 Siedlecka 2 Str., 72–010 Police, Poland

**Keywords:** skin, letrozole, estrogens

## Abstract

Skin is a target for hormones and a site of hormone production. Aromatase inhibitors such as letrozole reduce circulating estrogen. The aim of the study was to investigate the morphology of the dermis and immunoexpression of androgen receptor (AR), estrogen receptor α and β (ERα, ERβ), luteinizing hormone receptor (LHR), follicle-stimulating hormone receptor (FSHR), and cytochrome P450 aromatase (P450arom) in male rats with a deficit of estradiol. Experiments were performed on skin of 12 male rats. Rats in the experimental group received *per os* letrozole for 6 months. For morphological analysis, van Gieson, Sirius Red and orcein staining of sections was performed. In immunohistochemistry, reactions with specific antibodies (anti-P450arom, LHR, FSHR, ERα, ERβ) were used. In morphometric analysis, sections were stained with hematoxylin and eosin. Differences between groups were assessed by Mann-Whitney U-test. There were no differences in the diameter of collagen fibers. The dermis of letrozole-treated animals showed areas without collagen fibers, and expression of P450arom, ERα and ERβ was diminished in the skin of these animals. This study indicates that estrogens exert an effect via ERs that has a role in maintaining proper skin morphology in males, together with androgen. This is also the first documented expression of FSHR in the skin of male rats.

## I Introduction

Skin consists of three main parts: epidermis, dermis and hypodermis. The epidermis is a type of stratified squamous keratinized epithelium with keratinocytes as the main population of cells (90–95%), which create layers: stratum basale, spinosum, granulosum, lucidum (only in thick skin) and corneum. The dermis has two parts: papillary (loose connective tissue) and reticular layers (dense irregular connective tissue), and contains hydrated ground substance, fibers (collagen, elastic, reticular) and cells. Water, ground substance and fibers contribute to the thickness of the dermis. Human male skin is thicker than female skin at all ages [12]. Similarly, male mice have 40% thicker skin (and 190% thicker dermis) than females [2]. Regardless of sex, skin thickness decreases with age [12].

Skin plays many important functions for survival of organisms. The skin acts as a physical barrier that separates the mammalian organism from the external environment, so its primary function is protection from mechanical damage, temperature, micro-organisms, UV radiation and chemicals. This protective function of skin is associated with sophisticated sensory and signaling systems that can respond to environmental changes and signal to cells and organs in the body. The skin regulates several aspects of physiology, including body temperature and changes in fluid balance via sweat, and also acts as a reservoir for synthesis of vitamin D.

Human and animal skin also acts as an endocrine organ because it is a target for several hormones and neurotransmitters [71] and a site of hormone production [54, 72]. Epidermal keratinocytes and many cells in the dermis and skin appendages (sebaceous glands) express all necessary steroidogenic enzymes for *de novo* synthesis of sex steroids. Circulating androgens (dehydroepiandrosterone (DHEA) and androstenedione) are converted to testosterone and further to 5a-dihydrotestosterone (5a-DHT) by 5α-reductase type 2, which is mostly expressed in sebocytes and fibroblasts of the dermis [71]. Skin is also a source of estrogens because of the presence of cytochrome P450 aromatase (P450arom), which can convert androgens into estrogens, in human fibroblasts of the dermis [62].

Estrogens exert their effect by binding to specific estrogen receptors ERα and ERβ [33]. ERβ has been detected in epidermis, blood vessels, fibroblasts, hair follicles, dermal papilla cells, sebaceous glands and eccrine glands, while ERα is expressed in sebaceous glands, dermal papilla cells, eccrine glands, epidermis and outer sheaths of hair follicles [23, 27, 62]. The wide distribution of ERs in the skin is in agreement with studies showing that estrogens have many important and often protective functions in skin physiology [9, 46].

In humans, estrogens improve collagen content and quality, increase skin thickness and enhance vascularization [61]. The influence of estrogens on skin morphology is recognized in postmenopausal women, where wide changes in the content and quality of the dermal fibrous proteins and the ground substance of extracellular matrix (ECM) are present. These elements are responsible for the support and elasticity of the skin [30]. Types I and III collagens are by far the most abundant proteins in the dermis [14, 30].

Estrogens affect skin thickness and skin moisture by increasing glycosaminoglycans (GAGs), such as hyaluronic acid, and by increasing collagen production in the dermis. The presence of estrogens helps to maintain epidermal thickness and allows skin to remain hydrated and wrinkle-free [67]. Similar beneficial effects of estrogens have been recognized in animal skin, in which they stimulate the synthesis, maturation and turnover of collagen in rats and guinea pigs, and increase synthesis of hyaluronic acid in mice skin [16, 56, 57, 61].

Aromatase inhibitors such as anastrozole, letrozole and exemestane reduce circulating estrogen and are used in treatment of ER-positive breast cancer [20]. Hypoestrogenism in postmenopausal women causes the skin to become thinner with decreased collagen content, decreased elasticity, and increased wrinkling and dryness, which causes typical age-related deterioration of the skin [64]. A decrease of estrogens also reduces the number of blood vessels in the dermis, resulting in pale skin with insufficient nutrients moving to the skin surface [67]. Interestingly, a hypoestrogenic state is also associated with deficiency of TGF-β1, which causes scars to be less visible than at a higher level of estrogens [69].

The influence of a decrease of estrogens in skin in women and female animals (in contrast to males) has been widely reported. The aim of this study was to investigate the morphology of dermis elements and immunoexpression of androgen receptors (AR), ERα and ERβ, receptors for luteinizing hormone (LH-R) and follicle stimulating hormone (FSH-R), and aromatase cytochrome P450 (P450arom) in adult male rats with a long-term estradiol deficit.

## II Materials and Methods

### Animals and study design

Sexually mature 3-month-old male Wistar rats were maintained under standard conditions of lighting (12L:12D) and nutrition. The animals were randomly divided into control and experimental groups (6 rats per group). Rats in the experimental group received *per os* letrozole (Femara^®^; Novartis Pharma, Nuremberg, Germany), a non-steroidal inhibitor of P450arom, at a dose of 1 mg/kg b.w./day for 6 months, as described in detail elsewhere [25]. Animals were then sacrificed under thiopental anesthesia (120 mg/kg b.w., i.p., Biochemie GmbH, Vienna, Austria). The experiment was conducted in full accordance with Polish law and with the approval of the Ethics Committee of Pomeranian Medical University, Szczecin.

### Morphological analysis and immunohistochemistry

Dorsal and abdominal skin sections from control and experimental rats obtained after the dosing period were fixed by immersion in freshly prepared 4% paraformaldehyde and embedded in paraffin using a routine procedure. For morphological analysis, a series of sections (3–5 μm) of skin were mounted on glass slides and stained with hematoxylin and eosin (H-E). To visualize collagen organization, slides were subjected to van Gieson and Sirius Red 80 (Sigma Aldrich; 0.1% of Sirius Red in saturated aqueous picric acid) staining. Silver impregnation was performed to visualize reticular fibers, and elastic fibers were identified with orcein (Bio-Optica, Milan, Italy).

P450arom, AR, LH-R and FSH-R in skin tissues were detected immunohistochemically with specific primary antibodies: mouse monoclonal anti-P450arom (1:100, MCA 2077T, Serotec Ltd., Kidlington, Oxford, UK), mouse monoclonal antibody anti-AR (1:50, Clone AR441, Dako Corp., Carpinteria, CA, USA), rabbit anti-LHR polyclonal antibody (1:100, Santa Cruz Biotechnology, Santa Cruz, CA, USA; cat. no. sc-25828), and FSH-R rabbit anti-FSHR polyclonal antibody (H-190): Sc-13935 (1:100; Santa Cruz Biotechnology).

Deparaffinized sections were microwaved in citrate buffer (pH 6.0) for heat-induced epitope retrieval. After slow cooling to room temperature, the slides were washed in PBS twice for 5 min and then incubated for 60 min with the above primary antibodies. Next, slides were stained with avidin-biotin-peroxidase system with 5,5'-diaminobenzidine (DAB) as the chromogen (EnVision^+^System-HRP, code K4010, DakoCytomation, Glostrup, Denmark). Sections were washed in distilled H_2_O and counterstained with hematoxylin, excluding slides with nuclear localization of AR. As a negative control, the specimens were processed in the absence of the primary antibodies. Positive staining was defined by visual identification of DAB brown pigmentation under a light photomicroscope (Zeiss, Axioscope, Jena, Germany).

### Immunofluorescence study

ERα and ERβ in cells of skin tissue were detected using immunofluorescence, following incubation with specific primary antibodies: mouse monoclonal anti-ERα (1:50; F-10, or polyclonal rabbit anti-ERβ (1:50, H-150, both Santa Cruz Biotechnology). This was followed by treatment of slides with secondary antibodies conjugated with fluorochromes: anti-mouse IgG conjugated with FITC (1:64; Sigma-Aldrich, St. Louis, MO, USA) for ERα visualization or anti-rabbit IgG conjugated with Texas Red (1:100; Vector Labs. Burlingame, CA, USA) for ERβ visualization. After embedding, sections were evaluated using a confocal microscope (FV500, Olympus, Tokyo, Japan). For negative controls sections, the primary antibody was replaced by phosphate-buffered saline.

### Morphometry

Sections of dorsal and abdominal skin of control and experimental rats stained with H-E were analyzed to determine the diameters of collagen bundles in the reticular layer of the dermis. Morphometric measurements were obtained using Axio Vision Rel. 4.6 (Zeiss, Axioscope System, Germany).

### Statistical analysis

Data were analyzed using Statistica 5.0 PL for Windows (StatSoft, Poland). The sample size (n), median (M), arithmetical mean (X), standard deviation (SD), upper quartile and lower quartile (Q1–Q3) were calculated. Differences between groups were evaluated by non-parametric Mann-Whitney U-test because the distributions deviated from a normal Gaussian distribution in a Shapiro-Wilk test. The level of significance was p ≤ 0.05.

## III Results

### Morphological study

**Fig. 1.**
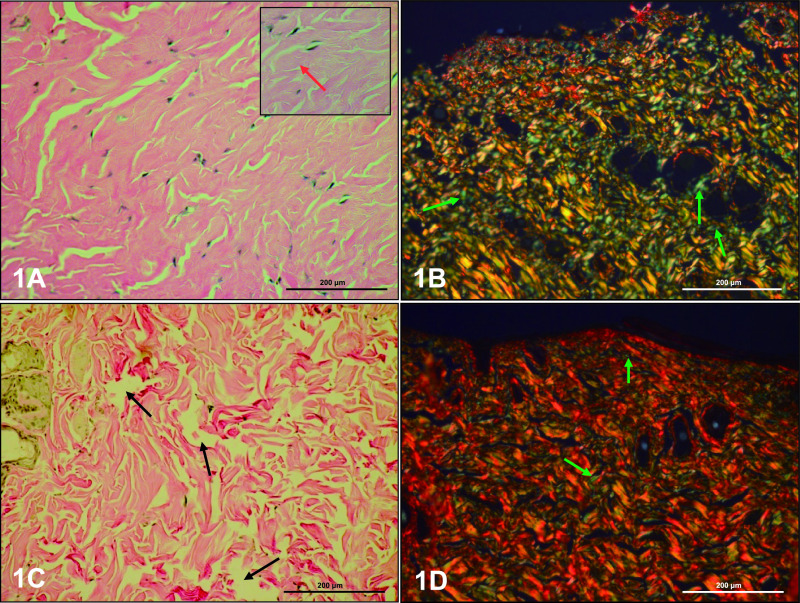
Collagen fibers in dorsal skin of control (**A, B**) and letrozole-treated (**C, D**) rats. Flattened nuclei were found in fibrocytes along and between collagen fibers (**A** insert, red arrow). The dermis of letrozole-treated rats was free of collagen fiber spaces (**C**, black arrow). Reticular fibers are indicated by green arrows (**B, D**). Staining: van Gieson (**A, C**), picrosyrius red (**B, D**). Magnification: **A–D** ×20; insert ×40.

**Fig. 2.**
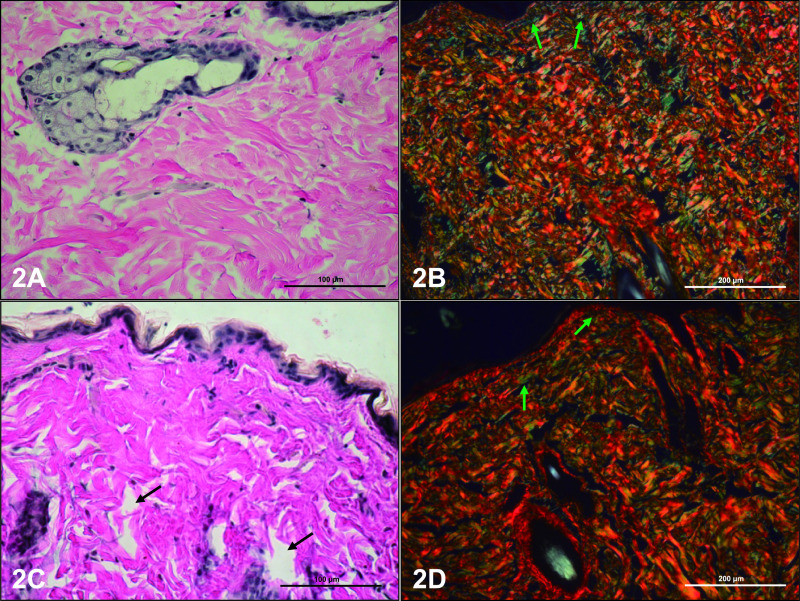
Collagen fibers in abdominal skin of control (**A, B**) and letrozole-treated (**C, D**) rats. The dermis of letrozole-treated rats was free of collagen fiber spaces (**C**, black arrows). Reticular fibers are indicated by green arrows (**B, D**). Staining: van Gieson (**A, C**), picrosyrius red (**B, D**). Magnification: **A** ×40; **B–D** ×20.

Histological analysis included the dermis of dorsal and abdominal skin in control and experimental rats. The collagen fiber composition was typical in the dorsal and abdominal dermis (Fig. 1A, B; Fig. 2A, B) in the control group. In the papillary dermis of controls, collagen fibers were located parallel to the epidermal surface and flattened nuclei of fibrocytes and other connective tissue cells were located along and between the fibers. In the reticular dermis, collagen fibers formed closely packed, thick woven bundles, between which flattened nuclei of cells were visible (Fig. 1A; Fig. 2A). There were no differences in the localization of collagen fibers in skin of experimental rats, but a few organizational changes were noted. The papillary dermis of letrozole-treated rats showed free collagen fiber spaces, and collagen fibers were defragmented in the reticular layer of these rats (Fig. 1C; Fig. 2C).

Collagen organization in the dermis was studied using picrosirius red staining and observed under polarized microscopy. According to Mesher (Junqueira’s Basic Histology. 14th ed. 2016), birefringence is highly specific for collagen. Collagen type I presents as a yellow, orange or red color, whereas collagen type III appears as green. Larger collagen fibers with yellow-reddish strong birefringence were assigned as collagen type I, while collagen type III displayed a greenish color and weak birefringence. The color and intensity of birefringence are due to a difference in patterns of physical aggregation and thickness of collagen fibers [35]. Thin collagen fibers have green to greenish-yellow polarizing colors (type III), whereas thick fibers appear as yellowish-orange through orange to red (type I).

In the papillary dermis of dorsal skin of control rats, thin collagen bundles with typical organization showed yellowish-orange birefringence, and a small amount of fibers with a greenish color were also observed (Fig. 1B). Thicker collagen bundles in the reticular dermis presented as regularly ordered structures with multiple orientations. The intense birefringence was different and varied from greenish-yellow to yellowish-orange. A small amount of thin fibers showed a green color (Fig. 1B). The papillary dermis of dorsal skin in experimental rats was dominated by collagen bundles with birefringence of yellowish-orange to reddish-yellow. Collagen bundles in the reticular dermis were regularly ordered with multiple orientations with different intensities of birefringence from yellowish-orange to reddish-yellow. Many fibers with a green color were visible on the surface of collagen bundles (Fig. 1D).

The papillary dermis of abdominal skin in control rats contained thin collagen bundles with a yellowish-orange color. Fibers with green birefringence were also observed (Fig. 2B). In the reticular dermis, collagen bundles with different intensities of birefringence of yellowish-orange to yellow-reddish were visible (Fig. 2B). Similarly to the controls, both the papillary and reticular dermis in abdominal skin of experimental rats were filled with well-organized collagen bundles with strong intensity birefringence of a yellow-reddish color, and thin fibers with a green color located on the surface collagen bundles (Fig. 2D).

**Fig. 3.**
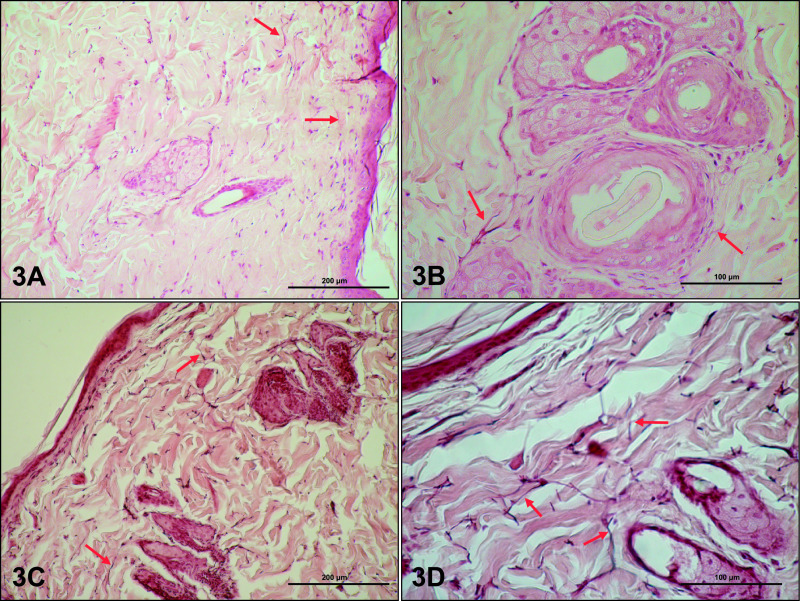
Elastic fibers (red arrows) in control (**A**: dorsal, **B**: abdominal skin) and letrozole-treated (**C**: dorsal, **D**: ventral skin) rats. Staining: orcein. Magnification: **A, C** ×20; **B, D** ×40.

Elastic fibers were visible mainly in the reticular layer of the dermis in both groups, either parallel or perpendicular to the epidermis (Fig. 3). These thin dark fibers formed a network between the collagen bundles. Elastic fibers in the dorsal and ventral skin dermis seemed to be decreased in particular areas in letrozole-treated rats, and the diameter of elastic fibers in the dermis of these rats appeared to be thicker than in the control group (Fig. 3C, D).

The localization of reticular fibers after silver impregnation (data not shown) was the same as that observed after staining with picrosirius red (green polarized color).

### Morphometry

Control and letrozole-treated rats had no significant differences in thickness of collagen fibers in the dorsal (6,963 ± 2,143 μm vs. 6,891 ± 2,158 μm) and abdominal (6,592 ± 2,403 μm vs. 6,639 ± 2,202 μm) skin (Table 1).

### Immunohistochemical (IHC) study

IHC reactions were used to identify the localization of AR, P450arom, ERα, ERβ, LH-R, and FSH-R, as described in the following sections. A summary of IHC results for expression of these proteins in skin in control and letrozole-treated rats is given in Table 2.

### Androgen receptor (AR)

**Fig. 4.**
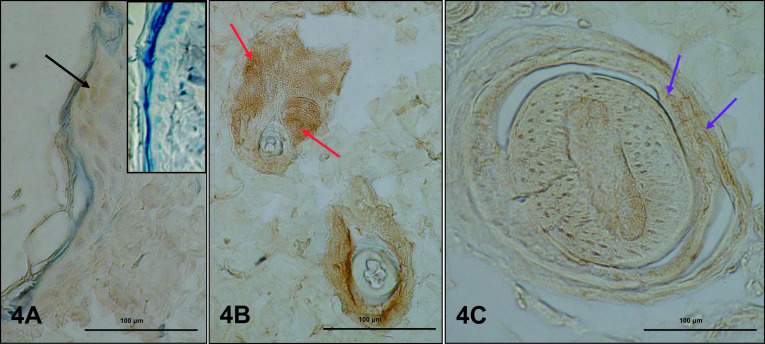
Immunoexpression of AR in abdominal skin of control rats. Immunopossitive reactions in nuclei of epidermal keratinocites (**A**, black arrow), sebocytes of sebaceus glands (**B**, red arrows) and keratinocytes of root sheath of hair (**C**, violet arrows); **A** (insert) is a negative control. IHC: **A** (and insert), **B, C** ×40.

AR was identified in cells of dorsal and abdominal skin in both control and experimental rats. In controls, AR was present in nuclei of keratinocytes of various layers of the epidermis (Fig. 4A). The product of the IHC reaction was clearly visible in the cytoplasm of sebocytes in the basal layer of the gland and in differentiated cells (Fig. 4C). Nuclei of cells of internal and external root sheaths of hair follicles and fibroblasts of the dermis were also AR-positive (Fig. 4B). There were no differences in localization or level of AR expression in letrozole-treated rats compared to controls.

### Cytochrome P450 aromatase (P450arom)

**Fig. 5.**
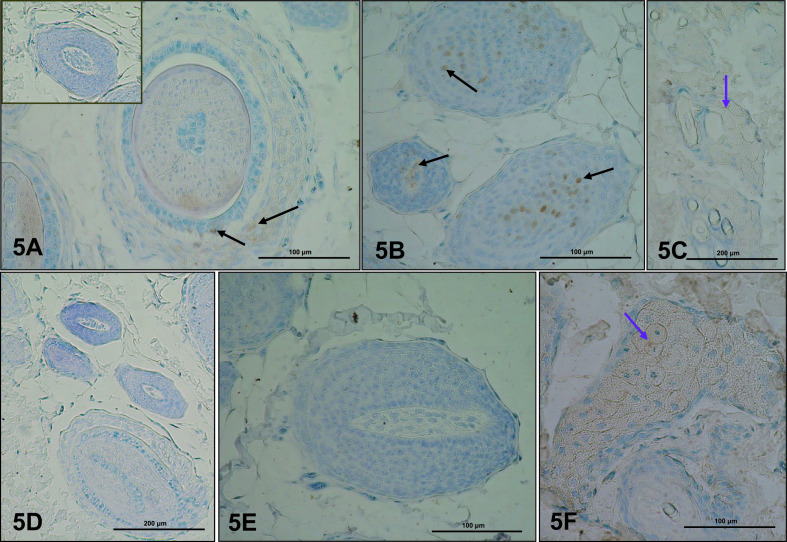
Immunoexpression of P450arom in dorsal skin of control (**A–C**) and letrozole-treated (**D–F**) rats. Immunoexpression was visible in cells of hair follicles (**A, B**, black arrows), and sebocytes of sebaceaus glands (**C, F**, violet arrow). **A** (insert) is a negative control. IHC: **A, B, E, F** ×40; **C, D** ×20.

P450arom was localized in the cytoplasm of cells in the dermis of dorsal and abdominal skin. In control rats, an immunopositive reaction occurred in cells of hair follicles (Fig. 5A, B). Sebocytes also displayed a weak reaction (Fig. 5C). There were no immunopositive fibroblasts in the evaluated specimens. In the dermis of experimental rats, P450arom was identified only in the cytoplasm of sebocytes (Fig. 5F), but the reaction was stronger than in controls. No expression of P450arom was detected in cells of hair follicles (Fig. 5D, E).

### Estrogen receptors α and β (ERα and ERβ)

**Fig. 6.**
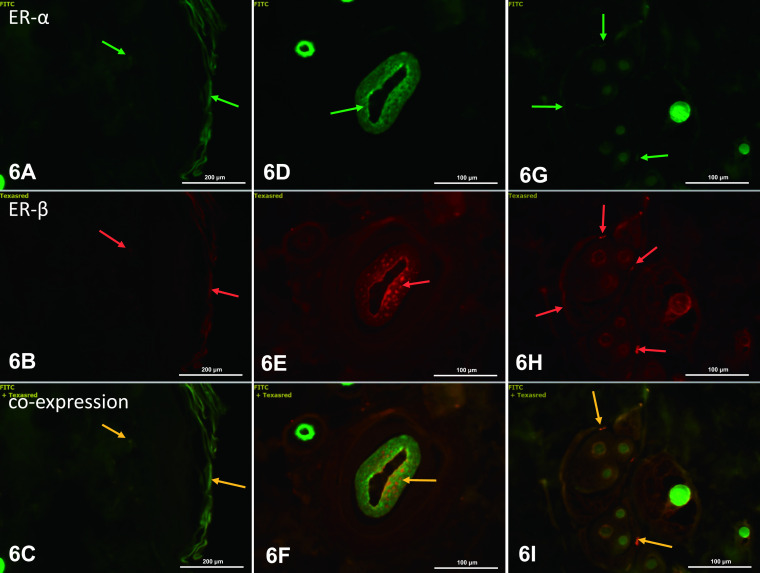
ERα and ERβ in the dorsal skin of control rats. Fluorescence of ERα was visible in keratinocytes of the stratum corneum and in cells of the dermis (**A**), and in keratinocytes of the internal root sheath (**D**) and sebocytes (**G**) (green arrows). Weak fluorescence for ERβ was seen in epidermal cells and in cells of the dermis (**B**), internal and external root sheath (**E**) and sebocytes (**H**) (red arrows). Orange arrows indicate coexpression of ERα and ERβ (**C, F, I**). IHC: **A–C** ×40; **D–I** ×20.

In the analysis of ERs, green and red fluorescence indicates ERα and ERβ, respectively, and coexpression of the two receptors appears as yellow/orange fluorescence. In dorsal skin of control rats, weak green (ERα, Fig. 6A), red (ERβ, Fig. 6B), and yellow (coexpression ERα/ERβ, Fig. 6C) fluorescence was present in the *stratum corneum* of the epidermis. In the dermis, single fibroblasts had very weak fluorescence indicating the presence of ERα (Fig. 6A), ERβ (Fig. 6B) and colocalization of the receptors (Fig. 6C). However, strong fluorescence was observed in keratinocytes of root sheaths. ERα expression was observed in nuclei of cells of the internal root sheath (Fig. 6D), whereas ERβ was found in cells of the internal and external root sheaths (Fig. 6E), with coexpression mainly in cells of the internal root sheath (Fig. 6F). Weak fluorescence for ERα was observed in nuclei of basal sebocytes and in the cytoplasm of differentiating sebocytes (Fig. 6G). Similar localization was observed for ERβ (Fig. 6H), and ERα and ERβ were mainly expressed in the same types of cells (Fig. 6I).

**Fig. 7.**
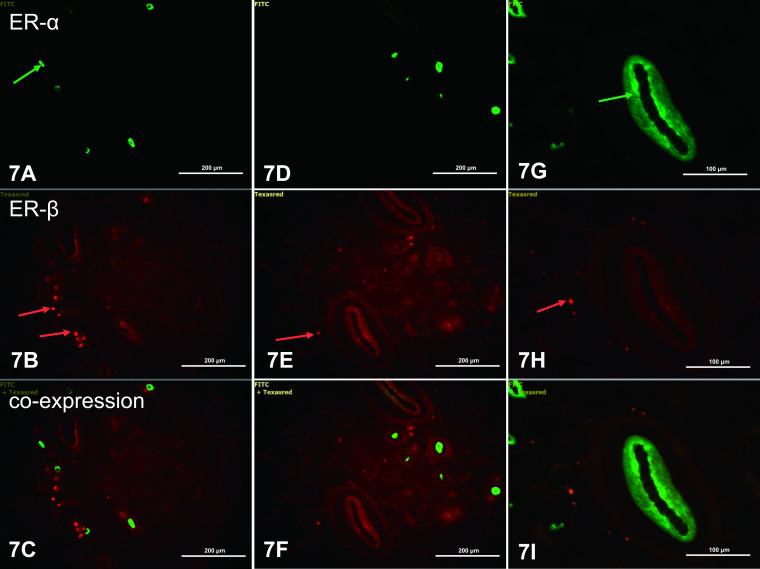
ERα and ERβ in abdominal skin of control rats. ERα fluorescence was seen in fibrocytes (**A**) and cells of the internal and external root sheath (**G**) (green arrows). ERβ fluorescence was found in many fibrocytes of the dermis (**B, E, F**, red arrows). There was no coexpression of ERα and ERβ (**C, F, I**). IHC: **A–F** ×20; **G–I** ×40.

In abdominal skin, expression of ERα and ERβ was not observed in keratinocytes of the epidermis. In the dermis, green ERα fluorescence was visible in the nuclei and cytoplasm of single fibrocytes (Fig. 7A). ERβ was found as the main ER in cells of the dermis (Fig. 7B, E, H). Colocalization of the two ERs was not observed in dermal cells (Fig. 7C, F, I). A weak green ERα fluorescence was seen in cells of the internal root sheath (Fig. 7G).

**Fig. 8.**
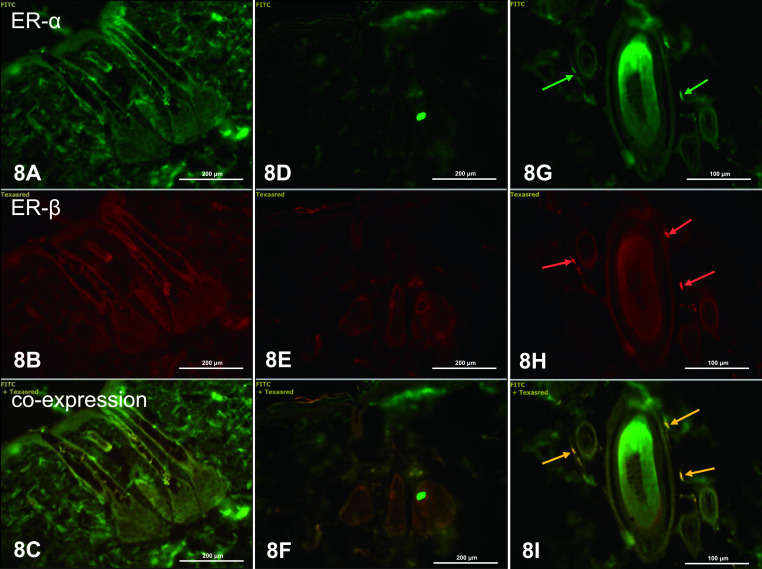
ERα and ERβ in the dorsal skin of letrozole-treated rats. There was a lack of ERα expression in keratinocytes of the epidermis (**A**) and sebocytes (**D**), and weak fluorescence in cells of the dermis near the connective tissue capsule of the root sheath (**G**, green arrow). Orange arrows indicate coexpression of ERα and ERβ (**C, F, I**). IHC: **A–F** ×20; **G–I** ×40.

**Fig. 9.**
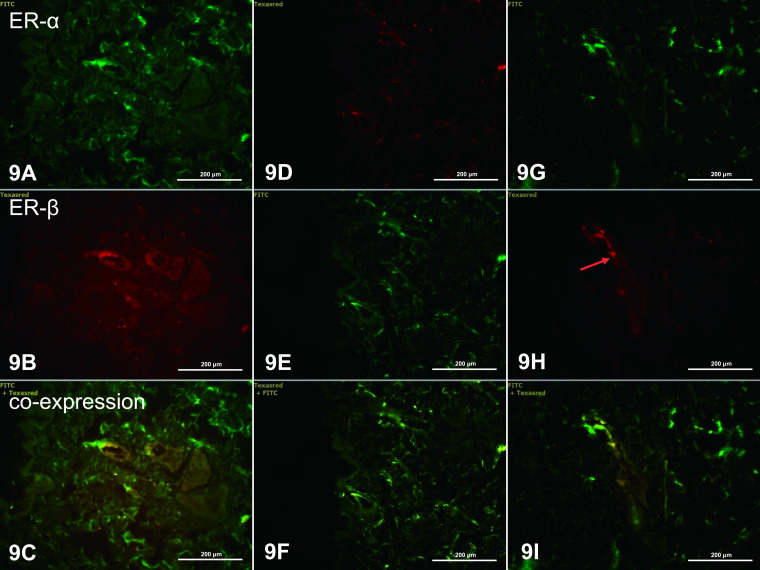
ERα and ERβ in the abdominal skin of letrozole-treated rats. Immunofluorescence of ERβ is visible in fibrocytes of the dermis (**H**, red arrow). IHC: **A–I** ×20.

In dorsal and abdominal skin of letrozole-treated rats, weak or no fluorescence was seen in cells of the epidermis and dermis (Figs. 8 and 9), which indicates decreased expression of ERs in all skin cells in letrozole-treated rats compared to controls. Expression was found only in nuclei of cells of the dorsal dermis, indicating ERα (Fig. 8G), ERβ (Fig. 8H) and coexpression of ERα/ERβ (Fig. 8I), particularly in the dermal root sheath of the hair.

### LH receptor (LH-R) and FSH receptor (FSH-R)

**Fig. 10.**
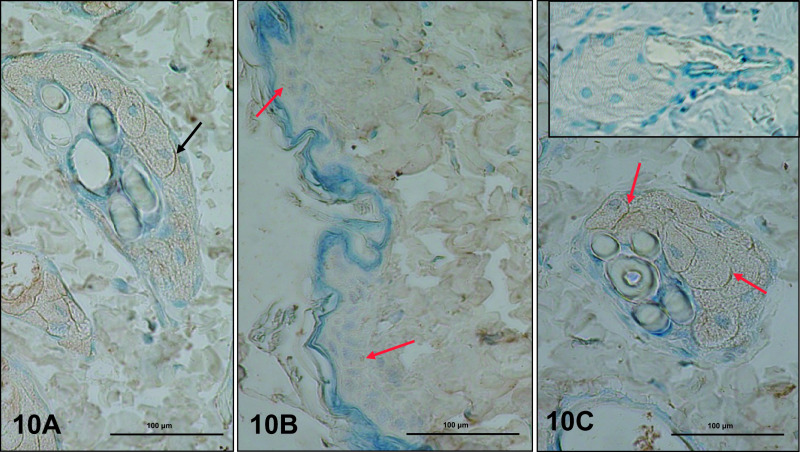
Immunoexpression of LH-R (**A**) and FSH-R (**B, C**) in skin of controls. LH-R was found in the plasma membrane of differentiating sebocytes (**A**, black arrows) and FSH-R in the plasma membranes of epidermal keratinocytes (**B**, red arrow) and differentiating and differentiated sebocytes (**C**, red arrows). **C** (insert) is a negative control. IHC: **A, B, C** ×40.

In dorsal and abdominal skin, immunoexpression of LH-R was observed only in the plasma membrane of sebocytes (Fig. 10A), with similar intensity in control and letrozole-treated rats. Weak immunoexpression of FSH-R was present in keratinocytes of the epidermis (Fig. 10B), with stronger expression in the plasma membrane of sebocytes (Fig. 10C) in dorsal and abdominal skin of control and letrozole-treated rats.

## IV Discussion

This study was performed to investigate the effect of long-term estrogen deficiency caused by letrozole (a nonsteroidal inhibitor of aromatase cytochrome P450) on skin morphology in adult male rats. Aromatase inhibitors can reduce the serum estradiol concentration, and are used in treatment of many disorders [1, 17, 22, 26, 37, 40, 50, 52, 65]. The influence of a decrease of estrogens on skin in women and female animals has been widely described. For example, Jackson *et al.* [21] showed that endocrine function changes with age and that estrogens protect skin from aging in females. In postmenopausal women, the level of 17-beta-estradiol (E2) decreases and oscillates between 15–60 pg/mL [18], and many typical features of skin aging occur in this population [4, 5, 58].

The effect of estrogens on female cutaneous physiology is well characterized, but the role of these hormones in males is poorly understood [13]. In men, 80% of plasma E2 is produced in peripheral tissues [24]. The 17-beta-estradiol level oscillates in the same range as that in postmenopausal woman [18]. However, conversion of testosterone to E2 by aromatase results in healthy older males having higher levels of circulating E2 than postmenopausal women. This is also associated with older males having increased adipose tissue, in which aromatase is expressed [3]. We have previously shown a significant reduction (43%) in serum 17β-estradiol (E2) [25] with no significant difference in the level of circulating testosterone after treatment of adult male rats with letrozole. Therefore, the changes in morphology in the skin in this study confirm a role for estrogens in normal male skin function. However, a decrease of estrogens did not cause changes in collagen organization and only resulted in small changes in elastic fibers structure in the dermis of the dorsal and abdominal skin.

In morphological analysis, we did not notice differences in localization, but a few organizational changes were found. The papillary dermis of letrozole-treated animals showed areas without collagen fibers, while collagen fibers in the reticular layer of these rats were defragmented. Statistical analysis of collagen-bundle diameters in the dermis in control and experimental rats showed no significant differences. Differences in organization of collagen fibers were clear in picrosirius red staining, which is sensitive and specific for types I and III collagen fibers [35]. Very well organized bundles of type I collagen and a rich amount of type III collagen were observed in the dorsal skin of experimental rats compared to controls. However, no differences were observed in abdominal skin.

It is well established that estrogen depletion results in decreased dermal collagen content in female animals [11, 31, 38]. The influence of gonadal hormones on collagen accumulation of male skin was examined in gonadectomized (GDX) animals. Laubach *et al.* showed that orchiectomy of mice resulted in almost undetectable testosterone levels [29]. Treatment with E2 did not cause a change in dermal thickness. However, there is evidence for involvement of androgens in connective tissue synthesis regulation [31, 32]. Markiewicz *et al.* suggested that estrogens may not be directly involved in regulation of collagen synthesis, but can play a role in regulating organization and stability of collagen fibrils [31]. However, in the adult male rat dermis, topical estriol cream applied on the dorsal skin caused increased thickness of collagen fibers [60]. Studies in male mice indicated that estrogens play a significant role in regulation of epidermal thickness, while dermal thickness is regulated by androgens [2]. This control of dermal thickness by androgens (not estrogens) and the direct correlation of dermal thickness with dermal collagen [53] may explain why we did not observe differences in localization and thickness of collagen fibers in the dermis of experimental animals compared to controls.

Elastic fibers were visible mostly in the reticular layer of the dermis in both groups of animals, and were localized parallel or perpendicular to the epidermis. The amount of elastic fibers in the dermis of letrozole-treated rats seemed to be higher than that in controls.

Most androgens and estrogens in men and women are synthesized locally by peripheral target tissues such as skin. Androgens act through ARs, which are located in different layers of keratinocytes in the epidermis and in keratinocytes of follicular dermal papillae, sebocytes, sweat glands, dermal fibroblasts, dermal papilla cells, endothelial cells and genital melanocytes [23, 42]. There are differences between males and females in expression of ARs in the skin [49]. In the present study, expression of ARs in controls was present in the population of cells listed above. No changes in expression or localization of ARs was found in skin of rats treated with letrozole, which may be associated with the very small but measurable increase of blood testosterone, in agreement with previous findings [28].

Androgens may also be converted to estrogens by P450arom in skin. In IHC staining, P450arom was present in follicular dermal papilla cells, in hair keratinocytes of internal and external root sheaths, and in sebocytes in control rats. This localization of P450arom expression is consistent with other studies [48, 49]. We did not observe immunopositive fibroblasts in the examined specimens, which may be due to dermal fibroblasts converting androgens into estrogens [10].

Long-term letrozole treatment caused termination of the activity of P450arom in almost all cell populations. Positive immunoexpression of P450arom was present only in sebocytes. Lack of expression in other populations of skin cells suggests that expression of P450arom is controlled by estrogens. P450arom activity in tissues other than skin is regulated by both androgens and estrogens [47, 70] or by glucocorticoids [34, 63], and regulation of P450arom expression in skin may differ between males and females [44].

Skin is not only a site of estrogen synthesis, but a target organ for hormones because of the presence of ERα and ERβ, which transduce estrogenic signals and are expressed in the skin of men, women and animals [23, 36, 71]. However, their localization in skin is not obvious [42]. In our experiments, ERs were expressed both in the dorsal and abdominal skin in control rats. ERβ was the main mediator of estrogen action in abdominal skin, which suggests that the distribution of ERs differs in different parts of the body. Immunolocalization of ER in our findings was not completely consistent with other studies, which have shown strong expression of ERβ in keratinocytes in all layers of the rat epidermis [41]. Differences in immunolocalization of ERs in epidermal cells may be caused by differences between the species, sexes, and even types of applied antibodies. However, we noticed coexpression of ERα and ERβ in dermal fibrocytes of the dorsal skin and expression of ERβ in the abdominal skin, in agreement with previous results [15, 42]. The presence of ERs in sebocytes and in keratinocytes in root sheath in our data is also in agreement with previous studies [23, 42]. In letrozole-treated rats there was decreased or even no Es expression, which suggests that ER expression is under control of estrogens. However, regulation of ER expression in skin may be more complicated [7, 45]. It is possible that diversified regulation of ER expression also occurs in skin, especially because locally produced steroids may control skin functions in humans and animals. Local formation of sex steroids provides autonomous control for target tissues, with adjustment of the formation and metabolism of sex steroids according to local needs [28].

In men, LH/hCG and FSH receptors have been extensively studied in their classical target organs: the testis and epididymis [59]. IHC analysis in our study showed the receptors for LH and FSH in dorsal and abdominal skin of control and experimental rats. LHR immunostaining was present in sebocytes, in partial agreement with other studies [39, 66, 68]. We found expression of FSHR in the epidermis and sebocytes. Previously, the presence of FSHR was noted only in female and male canine skin in the epidermis and adnexa [68]. Tests for FSHR expression in normal human skin showed no labelling in epidermal structures or in the dermis [66].

The function of gonadotropins in skin is not fully described, but the interaction between FSH and LH with their receptors stimulates the synthesis of testosterone, estrogens and progesterone, whose receptors have also been identified in human skin [42]. The presence of FSH-R and LH-R in skin of male rats indicates that synthesis and secretion of sex hormones may also be under the influence of gonadotropins.

The sebaceous gland exhibits expression of FSH, LH, P450arom, ERα, ERβ and AR, as shown in Table 1. Most sebaceous glands are present in association with a hair follicle, and together they are referred to as a pilosebaceous unit [55]. In our experiments, a decrease of estrogens caused disappearance of ERα and ERβ in sebocytes. There are a few theories on the function of the human sebum [43, 51, 55], but this material and the metabolic pathways regulating its composition and secretion rate are not completely understood, despite the large number of compounds that have been shown to regulate sebaceous gland function [43, 55].

The pilosebaceus unit is the main source of hormones in skin and expresses diverse hormone receptors to take up and interact with circulating hormones [6, 8]. Lutropin receptor expression indicates that androgen synthesis is under the influence of LH, while estrogen synthesis is under the influence of FSH. Several clinical studies have shown that androgens stimulate the sebaceous gland to produce sebum [19], whereas estrogens have the opposite effect of suppressing sebum production. The wide range of pilosebaceous unit activity and current clinical observations suggest that further studies are needed to understand the function and interaction of different hormones and hormone receptors.

In conclusion, male rat skin is an organ for which function is controlled by estrogens, as well as androgens. Our findings indicate that estrogens exerting effects via ERs can play a role with androgens in maintaining proper skin morphology in males. However, estrogen signaling in male skin is complex and further studies are required to understand the mechanisms. This study also provides the first evidence for expression of FSHR in skin of male rats.

## V Conflicts of Interest

The authors declare that there are no conflicts of interest.

## Figures and Tables

**Table 1.** Thickness of collagen fibers of dorsal and abdominal skin in control and letrozole-treated rats

Collagen fibers (μm)		Control	Letrozole
Dorsal skin	M	6,955	6,358
	Q_1_–Q_3_	5,43–8,28	5,318–8,145
	Mean ± SD	6,963 ± 2,143	6,891 ± 2,158
Abdominal skin	M	6,485	6,385
	Q_1_–Q_3_	4,685–8,425	5,24–7,82
	Mean ± SD	6,592 ± 2,403	6,639 ± 2,202

n = 6 animals/per group; n = 180/per group: number of collagen fibers in each group; M: median; Q_1_–Q_3_: upper quartile and lower quartile; SD: standard deviation.

**Table 2.** Localization of IHC staining indicating expression of AR, P450arom, ERα, ERβ, LH-R, and FSH-R in structures of skin in control (C) and letrozole-treated (Let) rats

	Epidermis			Dermal papilla			Root sheath			Sebocytes			Dermal cells	
	C	Let		C	Let		C	Let		C	Let		C	Let
AR	+	+		−	−		+	+		+	+		+	+
Arom	−	−		+	−		+	+		+	+		−	−
ERα	+/−	−		−	−		+	−		+	−		−	+/−
ERβ	+/−	−		−	−		+	−		+	−		−	+/−
LH-R	−	−		−	−		−	−		+	+		−	−
FSH-R	+	+		−	−		−	−		+	+		−	−

+: positive immunoexpression; +/−: moderate expression; −: lack of expression
